# Prevalence and influencing factors of non-suicidal self-injury among secondary school students seeking medical treatment

**DOI:** 10.3389/fpsyg.2025.1583373

**Published:** 2025-07-23

**Authors:** Xiaonv Fu, Mingzhe Zhao, Mingjin Luo, Chenxia Ye, Yating Wei, Janyue Cao, Haidong Song

**Affiliations:** ^1^Zhejiang Chinese Medical University, Hangzhou, China; ^2^Affiliated Mental Health Center and Hangzhou Seventh People's Hospital, Zhejiang University School of Medicine, Hangzhou, China; ^3^School of Mental Health and Psychological Sciences, Anhui Medical University, Hefei, Anhui, China; ^4^The Fourth School of Clinic Medicine, Tuandong Middle School, Nanyang, Henan, China

**Keywords:** secondary school student, non-suicidal self-injury, seeking-help, rate of consultation, influencing factors

## Abstract

**Background:**

Non-suicidal self-injury (NSSI) is a significant risk factor for suicide and has emerged as a growing public health concern among adolescents. Despite its severity, the consultation rate for NSSI among secondary school students remains notably low. While previous research has explored formal and informal help-seeking behaviors related to NSSI. In order to address this gap, this study aims to investigate the consultation rate and identify key factors associated with seeking medical treatment for NSSI.

**Methods:**

We conducted a cross-sectional study involving participants aged 12–20 years. The types and frequencies of NSSI were assessed using the Adolescent Self-Harm Questionnaire. Treatment-seeking behavior was evaluated through a self-developed questionnaire. Participants were recruited from both non-clinical settings (schools) and clinical settings (a tertiary hospital). Logistic regression analyses were employed to identify factors associated with seeking professional consultation for NSSI.

**Results:**

The consultation rate for NSSI was 15.64%. A total of 1,777 valid questionnaires were collected, including 1,586 non-clinical samples from secondary schools and 191 clinical samples. Factors positively associated with seeking medical treatment among secondary school students with NSSI included family awareness (*OR* = 10.452, 95%*CI* = 5.719–19.100, *p* < 0.001), being an only child (*OR* = 4.452, 95%CI = 1.847–10.730, *p* = 0.001), help-seeking behavior (*OR* = 2.694, 95%*CI* = 1.285–5.647, *p* = 0.009), older age (*OR* = 2.137, 95%*CI* = 1.630–2.801, *p* < 0.001), female (*OR* = 1.919, 95%*CI* = 1.002–3.676, *p* = 0.049), family income (*OR* = 1.699, 95%*CI* = 1.214–2.377, *p* = 0.002). No statistically significant associations were observed for educational stage, residential area, maternal education level, or maternal relationship in relation to treatment-seeking behavior.

**Conclusion:**

This study reveals a low consultation rate (15.64%) for NSSI among adolescents. Family awareness, being an only child, seeking help or not, age, and family income are factors influencing the seeking of medical treatment. Among these, family awareness has the greatest impact, followed by Seeking help or not. We appeal to parents to pay attention to the mental health of adolescents, build a good family relationship, and make efforts to promote the seeking of medical treatment.

## 1 Introduction

Non-suicidal self-injury (NSSI) is defined as the direct and intentional destruction of one’s own body tissues without lethal intent and without socially approved reasons (Cipriano et al., 2017). NSSI behavior is an increasingly serious clinical and public health issue that can have serious effects on teenagers’ health (Hawton et al., 2012). NSSI is quite prevalent among Chinese students (Qu et al., 2023; Lang and Yao, 2018; Yang and Feldman, 2018). Common NSSI methods include scratching, hitting, biting, hair-pulling, cutting, and pinching (Qu et al., 2023). Long-term and repeated self-injurious behaviors causing direct trauma to the body, are not conducive to teenagers’ physical and mental health and can cause a heavy burden to the family and social medical undertakings (Yong et al., 2023).

NSSI is regarded as a major public health problem among adolescents globally (Ma et al., 2021; Skegg, 2005) and is the greatest risk factor associated with future suicidal behavior (Ma et al., 2021). NSSI in adolescents has increased in recent years in China (Self-harm behaviors, suicidal ideation, and associated factors among rural left-behind children in west China, 2020). An epidemiological survey on NSSI among the Chinese population aged 5–25 shows that almost one in four (24.7%) students in primary, middle, high schools, and college reported NSSI behaviors in their lifetime (Qu et al., 2023). A survey in Nepal shows that as high as 44.8% of 327 adolescents reported a history of NSSI in the past year (Poudel et al., 2022), indicating a high incidence of NSSI (Tang et al., 2018). NSSI is prevalent among adolescents and carries a high risk of adverse outcomes. However, many adolescents with NSSI do not seek help from professional medical institutions (Hiew et al., 2024). A community-based study reported that only 10–13% of adolescents with NSSI sought medical treatment (Klineberg et al., 2013). Ystgaard’s survey on whether NSSI patients sought medical treatment showed that 18.8% received help from one or more health service institutions, 32.8% had no contact with healthcare services, and 48.4% received no help from anyone at all (Ystgaard et al., 2009). A cross-sectional survey study in 2023 on non-suicidal self-injury among left-behind children in China showed that only 22.0% sought professional psychological help, and 53.9% of left-behind children with NSSI received no treatment. Evidently, the treatment-seeking rate among adolescents with NSSI is low. Furthermore, compared with adults, children, and adolescents are less likely to seek medical help (Yong et al., 2023).

At the same time, these findings highlight the severity to which children with mental disorders fail to receive professional help (Sawyer et al., 2001). A randomized controlled trial showed that the intention to seek help improved among participants with emotional problems and suicidal ideation after receiving a brief, digital, online intervention (Han et al., 2023). An analysis of factors related to help-seeking among 121 adolescents who had sought medical treatment in Singapore in 2024 indicated that the severity of NSSI was associated with an increased likelihood of adolescents with NSSI seeking informal help from parents, friends, teachers, and other people. Gender and NSSI function were not related to help-seeking (Lustig et al., 2021). The above-mentioned studies did not report on the influencing factors of the treatment-seeking behavior of adolescents with NSSI. There is a scarcity of research on the treatment-seeking rate after NSSI and its related influencing factors. Moreover, the study conducted by Hiew et al. (2024) focused on hospitalized adolescents and pointed out the need to recruit from a larger non-clinical sample group. Therefore, this study recruited participants from secondary schools and clinical samples in mental health centers, obtaining consulted and unconsulted groups after NSSI occurrence.

Given the urgency of prompt treatment for both suicidality and NSSI, adolescents remained untreated for a significantly long period of time. The association of later treatment contact and higher symptom severity emphasizes the need to accelerate the help-seeking process (Lustig et al., 2021). Therefore, it is particularly necessary to study the influencing factors of seeking medical treatment for NSSI. The aim is to explore the relevant factors affecting treatment-seeking, thus providing a reference for policies to promote treatment-seeking among adolescents with NSSI.

## 2 Methods

### 2.1 Design and participant

In this study, a junior high school and a senior high school were randomly selected from a certain province in China. Using the cluster sampling method, a questionnaire survey was organized at the schools. At the outpatient and inpatient department of the Hangzhou Mental Health Center, a questionnaire survey was conducted on secondary school students who sought medical treatment after NSSI. Psychology teachers and postgraduate students served as investigators with unified instructions. Group tests were carried out by class. When the participants did not understand the items, the investigators provided objective explanations.

The diagnostic criteria recommended in the fifth edition of the Diagnostic and Statistical Manual of Mental Disorders (DSM-5) (Ammerman and Brown, 2018) were used to assess whether the patients had NSSI behaviors: having ≥3 NSSI behaviors in the past 6 months or ≥5 NSSI behaviors in the past year, with at least one NSSI behavior occurring in the most recent month. Throughout the process, these behaviors were not aimed at suicide and could not be explained by other physical or mental diseases. In this study, the concept of “consultation” has been explained to participants as going to hospital outpatient clinics, emergency rooms for wound treatment, or being hospitalized.

The sample size for the pre-post survey (Wang et al., 2024) was estimated using the formula *N* = *Z*^2^ × *σ*^2^[*P*(1-*P*)]/*d*^2^, where N represents the required sample size, *Z* is the confidence level (1.96 for 95% CI), *σ*^2^ = *P*(1-*P*) denotes the population variance, *P* is the estimated proportion set at 25%, based on prior NSSI consultation rates (Pfaff et al., 2001), and *d* is the margin of error (10%). Based on these parameters, the minimum required sample size was calculated to be 73 participants.

#### 2.1.1 Inclusion and exclusion criteria

Inclusion criteria: (1) Aged between 12 and 20 years old, regardless of gender; (2) Parents should consent to the questionnaire survey and sign the informed consent form; (3) Secondary school students who have engaged in self-harm are judged by at least two clinical doctors at the attending physician level or above to meet the DSM-5. Exclusion criteria: (1) Patients with comorbid major physical diseases and substance abuse; (2) Patients with intellectual disability, dementia, and severe cognitive impairment.

#### 2.1.2 Ethics approval and consent to participate

Both the student and their parents voluntarily participated in the study and signed written informed consent forms. This study has been approved by the Ethics Review Committee of the Seventh People’s Hospital of Hangzhou [Approval NO: Research (2024) Ethical Review (028)].

### 2.2 Measuring tools

#### 2.2.1 Socio-demographic information

A self-designed questionnaire was used to investigate demographic data, including the age, gender, residence (urban, rural, urban + rural), whether being an only child, parents’ marital status, parents’ educational level, relationship with parents, and family income of the study subjects.

#### 2.2.2 Adolescent self-harm questionnaire (ASHQ)

The ASHQ is a self-reported measure developed by Zheng Ying and then revised by Feng (2008) for assessing NSSI behavior in the past year. Adolescent Self-Injury Questionnaire. The NSSI’s method and the frequency were recorded, including 0 times, 1 time, 2–4 times, and 5 times or more (including 5 times). Assessment of the injury severity: A 5-level scoring system is used: none, mild, moderate, severe, and extremely severe. “None” means there is no damage to the skin; “mild” refers to minor damage to local tissues; “moderate” indicates damage to local tissues that requires treatment; “severe” means the injury require medical measures; “extremely severe” refers to the degree of physical harm that requires hospitalization. In the present study, the Cronbach’s *α* coefficient for the internal consistency of this scale was 0.940.

### 2.3 Statistical analysis

All analyses were conducted by SPSS (version 26.0) and SPSSAU software. The Kolmogorov–Smirnov test was employed for normality assessment. Measurement data that exhibited a normal distribution or were closely approximated to a normal distribution were presented as the mean ± standard deviation (x ± s). The data with a skewed distribution, it was characterized by the median and inter-quartile range [M(P25, P75)], and the Mann–Whitney U test was applied for comparisons between groups.

Descriptive statistics was conducted on the general information of the research subjects. Age, being a continuous variable, was analyzed using the independent-samples t-test and reported as (mean ± standard deviation). The Chi-square test was used for categorical variables, such as grade and residence. The variables demonstrating significant inter-group differences were designated as independent variables for multivariate logistic regression analysis. Whether to consult was used as the outcome variable. Aiming to explore the influencing factors associated with consultation behavior of secondary school students after NSSI. The significance level was set at a two-tailed probability of *p* < 0.05.

## 3 Results

### 3.1 Socio-demographic characteristics

A total of 1,576 valid questionnaires were collected from secondary schools (486 with NSSI). Regarding whether to seek medical treatment after NSSI, 211 people filled out the relevant part. Our study found that the consultation rate of NSSI was 15.64% (33/211), and 84.36% (178) did not seek medical treatment. Participants’ age ranged from 10 to 20 years, with an average age of (14.49 ± 1.52), Junior middle school students accounted for 70.81% (1,116/1,576), and senior middle school students accounted for 29.19%(460/1,576). Researchers collected 10 untreated cases through school psychological counseling in Hangzhou, alongside a non-clinical cohort of 1,586 participants.

Including 191 clinical samples, a total of 412 questionnaires were collected to assess factors related to medical treatment-seeking behavior. Among them, 54.37%(224) had sought medical treatment, and 45.63%(188) had not, with 70.39%(290) females, and 29.61%(122) males. The age ranged from 10 to 20 years, with an average age of (15.12 ± 1.92) years. Junior middle school students accounted for 58.98%(243/412), and senior middle school students for 41.02%(169/412).

### 3.2 Univariate analyses

#### 3.2.1 Socio-demographic and clinical characteristics

Table 1 presents the socio-demographic and clinical characteristics of the enrolled individuals. A total of 412 valid questionnaires were received regarding seeking medical treatment after NSSI. In the consulted group, 47.32% were junior middle school and 52.68% were senior middle school (*χ*^2^ = 27.582, *P*<0.001); 22.32% were boy, 77.68% were girl (*χ*^2^ = 12.517, *P* < 0.001); 29.46% were the only children, 70.54% were the non-only children (*χ*^2^ = 12.517, *P* < 0.001). In the consulted group, the proportion of always bullied by classmates were 27.23% (*χ*^2^ = 48.660, *p* < 0.001) (Table 1).

**Table 1:** Sociodemographic and clinical characteristics.

Variables	Category	Consulted	Unconsulted	*χ* ^2^ */t*	*P*
		*n* = 224(%)	*n* = 188(%)		
Learning stage	Junior middle school	106 (47.32%)	137 (72.87%)	27.582	<0.001
	Senior middle school	118 (52.68%)	51 (27.13%)		
Gender	Boy	50 (22.32%)	72 (38.30%)	12.517	<0.001
	Girl	174 (77.68%)	116 (61.70%)		
Age	12-20 year			8.907	<0.001
Only child	Yes	66 (29.46%)	14 (7.45%)	31.667	<0.001
	No	158 (70.54%)	174 (92.55%)		
Childhood residence area	Urban	117 (52.23%)	74 (39.36%)	8.774	0.012
	Urban and rural	69 (30.80%)	63 (33.51%)		
	Rural	38 (16.97%)	51 (27.13%)		
Bullying	None	71 (31.70%)	109 (57.98%)	48.660	<0.001
	Occasionally	92 (41.07%)	71 (37.77%)		
	always	61 (27.23%)	8 (4.25%)		
Psychological consulting	Yes	181 (80.80%)	140 (74.47%)	2.384	0.123
	No	43 (19.20%)	48 (25.53%)		
NSSI times	<5 times	22 (9.82%)	46 (24.47%)	107.934	<0.001
	5–10 times	19 (8.48%)	51 (27.13%)		
	10–20 times	29 (12.95%)	52 (27.66%)		
	20–30 times	45 (20.09%)	26 (13.83%)		
	≥30 times	109 (48.66%)	13 (6.91%)		
NSSI extent	No damage	21 (9.38%)	43 (22.87%)	95.765	<0.001
	Mild NSSI	80 (35.71%)	125 (66.49%)		
	Moderate NSSI	63 (28.12%)	19 (10.11%)		
	Severe NSSI	60 (26.79%)	1 (0.53%)		
NSSI types	One to five types	52 (23.21%)	121 (64.36%)	84.743	<0.001
	Six to ten types	75 (33.48%)	49 (26.06%)		
	More than ten types	97 (43.31%)	18 (9.58%)		

Among secondary school students after NSSI, the proportion of those with the number of NSSI more than 30 times was 48.66% (*χ*^2^ = 107.934, *p <* 0.001). The proportion for those with no, mild, moderate, and severe NSSI degrees were 9.38, 35.71, 28.12, and 26.79%, respectively, (*χ*^2^ = 95.765, *p <* 0.001) (Table 1).

### 3.3 Family factors

Regarding family factors, a series of chi-square tests were conducted to explore their associations with the medical-seeking behavior of NSSI middle school students (Table 2).

**Table 2:** Family factors on seeking medical treatment after NSSI.

Variables	Category	Consulted	Unconsulted	*χ* ^2^ */t*	*P*
		*n* = 224(%)	*n* = 188(%)		
Father’s educational level	Junior middle school or below	116 (51.79%)	105 (55.85%)	4.832	0.089
	Senior middle school or technical school	57 (25.45%)	56 (29.79%)		
	Collage or above	51 (22.76%)	27 (14.36%)		
Mother’s educational level	Junior middle school or below	124 (55.36%)	116 (61.70%)	8.385	0.015
	Senior middle school or technical school	51 (22.77%)	51 (27.13%)		
	Collage or above	49 (21.87%)	21 (11.17%)		
Parents’ marital status	married	170 (75.89%)	154 (81.91%)	2.207	0.137
	Single-parent families	54 (24.11%)	34 (18.09%)		
Relationship with father	Very good	31 (13.84%)	45 (23.94%)	11.630	0.020
	Good	52 (23.21%)	54 (28.72%)		
	General	100 (44.64%)	63 (33.51%)		
	Relatively poor	31 (13.84%)	18 (9.57%)		
	Very poor	10 (4.47%)	8 (4.26%)		
Relationship with mother	Very good	39 (17.41%)	69 (36.70%)	24.111	<0.001
	Good	76 (33.93%)	63 (33.51%)		
	General	82 (36.61%)	44 (23.40%)		
	Relatively poor	14 (6.25%)	5 (2.66%)		
	Very poor	13 (5.80%)	7 (3.73%)		
Monthly total family income (RMB)	<3,000	19 (8.48%)	34 (18.09%)	25.078	<0.001
	3,000–6,000	60 (26.79%)	72 (38.30%)		
	6,000–10,000	70 (31.25%)	53 (28.19%)		
	>10,000	75 (33.48%)	29 (15.42%)		
Family members know	Yes	172 (76.79%)	35 (18.62%)	138.343	<0.001
	No	52 (23.21%)	153 (81.38%)		
Help seeking	Yes	74 (33.04%)	31 (16.49%)	14.736	<0.001
	No	150 (66.96%)	157 (83.51%)		

In the consulted group, the proportion of patients whose family members were informed about their NSSI was 76.79% (*χ*^2^ = 138.343, *p* < 0.001); the proportion of students who sought help from family members, classmates, or teachers after NSSI was 33.04% (48.86%, *χ*^2^ = 14.736, *p* < 0.001) (Table 2).

Family income showed a significant positive correlation with medical consultation frequency. The impact on seeking medical attention after NSSI was statistically significant (*χ*^2^ = 25.078, *p* < 0.001) (Table 2).

The relationship between secondary school students and their fathers (*χ*^2^ = 11.63, *p* = 0.02) and mothers (*χ*^2^ = 24.111, *p* < 0.001) was found to have a statistically significant impact on medical treatment after NSSI (Table 2).

When it came to the mother’s education level, in the consulted group, the proportion of NSSI middle school students with mothers college-educated or above was 21.87% (*χ*^2^ = 8.835, *p* = 0.015). This shows that the mother’s education level has a statistically significant impact on the likelihood of NSSI middle school students seeking medical consultation (Table 2). For the parents’ education level, in the consulted group, the chi-square test demonstrated that the proportion of NSSI middle school students whose fathers had a college degree or above was 22.76% (*χ*^2^ = 4.832, *p* = 0.089).

Regarding the parents’ marital status, the chi-square test result (*χ*^2^ = 2.207, *p* = 0.137) indicated there is no statistical significance on whether NSSI secondary school students would seek medical treatment after NSSI. This suggests the parents’ marital status may not be a decisive factor in their medical-seeking behavior (Table 2).

Overall, these findings suggest that multiple family-related factors, such as parental education, family relationships, household income, and information-sharing within the family, are related to the medical-seeking behavior of NSSI secondary school students, which provides important insights for preventing and intervening in NSSI among adolescents.

### 3.4 Multivariate analysis

The results of logistic regression analysis revealed the influencing factors on seeking medical treatment after NSSI. Family members’ awareness (*OR* = 10.452, 95%*CI* = 5.719–19.100, *p* < 0.001), being an only child (*OR* = 4.452, 95%CI = 1.847–10.730, *p* = 0.001), seeking help (*OR* = 2.694, 95%*CI* = 1.285–5.647, *p* = 0.009), being bullied by classmates (*OR* = 2.533, 95%*CI* = 1.613–3.977, *p* < 0.001), age (*OR* = 2.137, 95%*CI* = 1.630–2.801, *p* < 0.001), gender (*OR* = 1.919, 95%*CI* = 1.002–3.676, *p* = 0.049), family income (*OR* = 1.699, 95%*CI* = 1.214–2.377, *p* = 0.002) were positively correlated with the medical treatment of secondary school students after NSSI. There were no statistically significant differences in the medical treatment of middle school students with NSSI in terms of learning stage (*OR* = 0.447, 95%*CI* = 0.191–1.045, *p* < 0.063), Residence (*OR* = 0.831, 95%*CI* = 0.628–1.099, *p* = 0.194), mother’s educational level (*OR =* 0.939, 95%*CI* = 0.609–1.449, *p* = 0.777), and relationship with mother (*OR* = 1.278, 95%*CI* = 0.934–1.773, *p* = 0.123) (Table 3).

**Table 3:** Binary logistic regression analysis for the influencing factors on seeking medical treatment after NSSI.

Variables	*β*	*z*	Wald *χ*^2^	*p*	*OR*	95% *CI*
Age	0.759	5.497	30.213	<0.001	2.137	1.630 ~ 2.801
Learning stage	−0.806	−1.859	3.456	0.063	0.447	0.191 ~ 1.045
Gender	0.652	1.966	3.866	0.049	1.919	1.002 ~ 3.676
Mother’s education level	−0.063	−0.283	0.080	0.777	0.939	0.609 ~ 1.449
Relationship with mother	0.252	1.542	2.376	0.123	1.287	0.934 ~ 1.773
Only child	1.493	3.327	11.070	0.001	4.452	1.847 ~ 10.730
Residence	−0.185	−1.298	1.686	0.194	0.831	0.628 ~ 1.099
Bulling	0.929	4.038	16.307	<0.001	2.533	1.613 ~ 3.977
Family income	0.530	3.091	9.556	0.002	1.699	1.214 ~ 2.377
Family members know	2.347	7.629	58.195	<0.001	10.452	5.719 ~ 19.100
Seeking help or not	0.991	2.625	6.888	0.009	2.694	1.285 ~ 5.647

## 4 Discussion

This study examined the consultation rate following NSSI and identified key factors influencing treatment-seeking behavior. Our results showed that the consultation rate among secondary school students after NSSI was 15.64%. While this rate was substantially lower than the 25% reported in a previous study (Pfaff et al., 2001; Sanci et al., 2010), it remains higher than the findings in Hasking’s research. In a study of 526 adolescents with a history of NSSI, only 6.6% of them sought help from mental health professionals (Hasking et al., 2015). The rate was also similar to that in a community-based study, where the rate of seeking medical treatment among adolescents with NSSI was 10–13% (Klineberg et al., 2013). The differences in the treatment-seeking rate may be related to the following factors: most researches used no uniform definition of NSSI (Tang et al., 2018), differences in the cultural background of the sample, and the use of different tools to assess the self-injuring method (Poudel et al., 2022). The above (Hasking et al., 2015; Klineberg et al., 2013) mentioned studies highlight that the treatment-seeking rate among adolescents with NSSI is low.

Factors influencing the seeking medical treatment of secondary students after NSSI include family awareness, family income, being an only child, seeking help, age, gender, and being bullied.

Among these, family awareness was found to the greatest impact. Medical help-seeking was more prevalent in adolescents with NSSI when families were aware versus unaware of their condition. When secondary school students with NSSI informed their parents, parental concerns about their children’s behavioral health problems may be a promising strategy to increase access to services (Ellingson et al., 2004). Moreover, family income is also an important influencing factor. Among adolescents engaging in NSSI, household income was identified as a significant predictor of medical health clinical consultation, showing a positive correlation with help-seeking behavior. Previous studies have shown that seeking help may be restricted by financial resources (Hiew et al., 2024).

Regarding seeking help, multivariate analysis showed that seeking help was a facilitating factor for consultation. The act of seeking help here may involve reaching various parties such as classmates, friends, teachers, and parents. Adolescents most frequently seek help from their peers, followed by their parents and professionals (Hiew et al., 2024). A study by Hasking suggested that disclosing NSSI to peers might assist in seeking help, but it could also exacerbate or encourage NSSI within the peer group. Conversely, confiding in adults might be an important protective factor. It can enhance adaptive coping skills and the belief in the ability to adopt alternative coping strategies to prevent suicide, and may also reduce the severity of NSSI (Hasking et al., 2015).

Only-child status was a significant factor influencing medical treatment among secondary students with NSSI. It is generally believed that only-child groups and non-only-child groups have different personality, cognition, and influence characteristics due to the influence of family environment (Li et al., 2013). Only-child receive too much attention and excessive praise from their parents and grandparents (Yang et al., 2017). Children who are only-child’s receive their families’ undivided attention (Wang et al., 2000). In families with an only-child, attention, time, and energy provided by parents of the only-child may lead to better parental guidance and individual care (Chi et al., 2020).

This study found that the seeking professional mental health service of secondary school students after NSSI was positively correlated with age. Age was a significant predictor of clinical service utilization. Emotional maturity comes with an increase in chronological age (Kusum et al., 2023). This might be because students in high school have a better understanding of their mental health status. It is also possible that the accompanying emotional problems have persisted for a longer time, increasing the likelihood of being noticed by parents.

This study found that after NSSI, female adolescents exhibited 3.48-fold higher odds of mental health clinical consultation compared to males. This may be related to the fact that the incidence of NSSI among females is higher than that among males (Tang et al., 2018). Additionally, a study by Kessler et al. suggested that the females are more willing to seek help for emotional problems (Kessler et al., 1981). Compared with the male, the female prefer to choose formal services more frequently (van den Toren et al., 2020). It could also be because females mature earlier than males and are more willing to communicate with their parents. However, some research has shown that after the most recent episode of NSSI, the proportion of males hospitalized was significantly higher than that of females (Ystgaard et al., 2009).

Among adolescents with NSSI, peer victimization was association with seeking medical treatment. This study suggest there is a strong link between bullying and NSSI (Myklestad and Straiton, 2021). Adolescents who are bullied may use self-harm as a form of calling for help (Heerde and Hemphill, 2019), this study indicates that there may be no direct causal relationship between bullying and seeking medical treatment. The relationship among them demands further research. Anyhow, bullied students need more attention from teachers, parents and society. The mediating effect of bullying victimization on the relationship between childhood trauma and NSSI (Xiao et al., 2020).

Regarding the clinical characteristics of NSSI, clinical consultation seeking-behavior were positively correlated with NSSI severity. This may be because those with more severe NSSI are more motivated to seek help (Hiew et al., 2024). Univariate analysis demonstrate significant between-group in NSSI characteristics including severity and frequency. Some studies suggest that adolescents who engage in NSSI and have access to health care services report more problems than those who receive no help (Ystgaard et al., 2009). Moreover, as the frequency of NSSI increases, with seasonal changes, it is more likely to be detected by family members or teachers. It may also indicate the exacerbation of accompanying emotional symptoms, which in turn attracts more family attention and promotes seeking medical treatment.

Regarding sample recruitment, participants were sourced from both non-clinical (school) and clinical (hospital) settings. As school-based participants who had sought medical care ultimately reintegrated into the community after clinical visits, we considered the treated populations from both sources to be comparable in nature, thus justifying their inclusion in comparative analyses.

## 5 Limitations

First, while this study provides initial insights, its regional sampling limits generalizability. Future nationwide surveys should assess geographic variations in prevalence, consultation rates, and associated factors. Additionally, uneven grade distribution in our cohort suggests the need for expanded sampling to reduce estimation errors in subsequent research. Moreover, data collection in school computer labs may have introduced selection bias, as some students potentially withheld information due to stigma-related concerns. Furthermore, our study cohort showed a slight female predominance, highlighting the need for gender-balanced sampling in future research. Finally, this study assessed depressive symptoms but did not include clinical diagnoses of psychiatric disorders.

To improve consultation rates among adolescents with NSSI, delivering mental health literacy initiatives for teacher、students and parents is critical to enhance recognition of NSSI, depression, and comorbid mental health conditions. Future mental health initiatives should foster tripartite alliances among schools, families, and local mental health centers. When teachers identify NSSI behaviors or psychiatric symptoms in students, prompt communication with parents-followed by coordinated referrals to partnered mental health facilities (with parental consent)-could streamline clinical intervention. Particular attention should be directed to vulnerable adolescents, including middle-school students, males, younger students, non-only children, and victims of bullying.

## 6 Conclusion

The study reveals a concerningly low rate of medical consultation among secondary school students following NSSI. Regarding the influencing factors of seeking medical treatment, the most significant is family awareness, followed by the only child, seeking help from the outside and age. Schools, families and society should pay more attention to younger middle-school students, especially junior high school students. We appeal to parents to pay attention to the mental health of adolescents, build a good family relationship, and make efforts to promote the seeking of medical treatment. In the school, teachers and other students should be inclusive and accepting of students with NSSI and other mental disorders, reduce their stigma, and create conditions for promoting medical treatment.

## Data Availability

Publicly available datasets were analyzed in this study. This data can be found at: 金山文档 | WPS云文档 data set: https://kdocs.cn/l/ckqtfSba4ZO9.
